# VIP Stabilizes the Cytoskeleton of Schlemm's Canal Endothelia via Reducing Caspase-3 Mediated ZO-1 Endolysosomal Degradation

**DOI:** 10.1155/2021/9397960

**Published:** 2021-09-13

**Authors:** Xiaotong Lou, Qianxue Mou, Bowen Zhao, Jingqiu Huang, Ke Yao, Zhaoxia Luo, Meng Ye, Yuanyuan Hu, Qiming Duan, Xing Li, Zheng Wen, Zhiqi Chen, Hong Zhang, Yin Zhao

**Affiliations:** ^1^Department of Ophthalmology, Tongji Hospital, Tongji Medical College, Huazhong University of Science and Technology, Wuhan 430030, China; ^2^Gladstone Institutes, San Francisco, 94145 California, USA; ^3^Department of Anesthesiology, Tongji Hospital, Tongji Medical College, Huazhong University of Science and Technology, Wuhan 430030, China; ^4^Division of Cardiology, Tongji Hospital, Tongji Medical College, Huazhong University of Science and Technology, Wuhan 430030, China

## Abstract

**Objectives:**

In glaucomatous eyes, the main aqueous humor (AH) outflow pathway is damaged by accumulated oxidative stress arising from the microenvironment, vascular dysregulation, and aging, which results in increased outflow resistance and ocular hypertension. Schlemm's canal (SC) serves as the final filtration barrier of the main AH outflow pathway. The present study is aimed at investigating the possible regulation of vasoactive intestinal peptide (VIP) on the cytoskeleton by stabilizing ZO-1 in SC.

**Methods:**

Model of chronic ocular hypertension (COH) induced by episcleral venous cauterization was treated with topical VIP. The ultrastructure of junctions, ZO-1 levels, and permeability of the SC inner wall to FITC-dextran (70 kDa) were detected in the COH models. The F-actin distribution, F/G-actin ratio, and ZO-1 degradation pathway in human umbilical vein endothelial cells (HUVECs) and HEK 293 cells were investigated.

**Results:**

ZO-1 in the outer wall of the SC was less than that in the inner wall. COH elicited junction disruption, ZO-1 reduction, and increased permeability of the SC inner wall to FITC-dextran in rats. ZO-1 plays an essential role in maintaining the F/G-actin ratio and F-actin distribution. VIP treatment attenuated the downregulation of ZO-1 associated with COH or H_2_O_2_-induced oxidative damage. In H_2_O_2_-stimulated HUVECs, the caspase-3 inhibitor prevents ZO-1 disruption. Caspase-3 activation promoted endolysosomal degradation of ZO-1. Furthermore, a decrease in caspase-3 activation and cytoskeleton redistribution was demonstrated in VIP + H_2_O_2_-treated cells. The knockdown of ZO-1 or the overexpression of caspase-3 blocked the effect of VIP on the cytoskeleton.

**Conclusion:**

This study provides insights into the role of VIP in stabilizing the interaction between the actin cytoskeleton and cell junctions and may provide a promising targeted strategy for glaucoma treatment.

## 1. Introduction

Primary open-angle glaucoma (POAG) is characterized by pathological ocular hypertension and visual field defects [1]. The exact pathogenesis of POAG remains unclear. Elevated intraocular pressure (IOP) is a well-established risk factor for the development and progression of glaucomatous optic neuropathy [2]. Aqueous humor (AH) is secreted by the ciliary body and finally drained through the trabecular meshwork- (TM-) Schlemm's canal (SC) pathway and the uveoscleral outflow pathway. Once the balance between AH production and outflow is disturbed, pathological IOP fluctuations occur. It is generally accepted that impaired AH outflow induced by increased resistance of the TM-SC pathway results in elevated IOP. In glaucomatous eyes, the TM-SC pathway is damaged by accumulated oxidative stress arising from the microenvironment, vascular dysregulation, and aging [3].

As the final filtration barrier of the AH outflow pathway, the inner wall of the SC plays a critical role in IOP regulation. It has been verified that the inner wall of the SC in glaucomatous eyes is stiffer than that in normal eyes, indicating an association between pathological changes in the biophysical characteristics of SC endothelia (SCE) and ocular hypertension [4, 5]. Drugs targeting Rho-kinase inhibition or actin depolymerization were recently introduced to reduce cell stiffness in the TM-SC pathway and, consequently, reduce IOP [6–8]. However, current antiglaucoma drugs have failed to manipulate the TM-SC pathway in clinic.

Finely organized semiflexible actin filaments determine cell stiffness [9]. It was verified that abnormal actin polymerization, such as stress fiber formation, may increase cell stiffness [10]. Dynamic interactions exist between the cytoskeleton and cell junctions, both of which regulate each other. During Rho/myosin light chain (MLC) activation, which induces blood-brain barrier disruption, sustained actin polymerization occurs with the disassembly of cell junctions [11]. Madara et al. observed rapid junction disassembly after the administration of an F-actin disrupting toxin in absorptive cells [12], suggesting that the redistribution of F-actin can be responsible for junction loss. In turn, junction instability elicits F-actin redistribution. Odenwald et al. reported that knockdown of the junction protein ZO-1 resulted in the accumulation of dense cytoplasmic F-actin in MDCK cells [13].

ZO-1 plays a vital role in mechanosensation, polarity, and adhesion [14, 15]. In addition, ZO-1 serves as a cytoskeletal connector that directly anchors F-actin to cell junctions with its actin-binding region and interacts with multiple other junctional components [16, 17]. Oxidative stress induced the dissociation of ZO-1 from junctions in epithelial and endothelial cells [18, 19]. However, it is not clear if ZO-1 of SCE changes in response to ocular hypertension and contributes to the disordering of F-actin.

Vasoactive intestinal peptide (VIP), which is composed of 28 amino acids, is a neurotransmitter, neurotrophic, or neuroprotective factor [20]. VIP was expressed around the SC, and the receptor of VIP (VPAC2) has been observed in SCE [21, 22]. In our previous study, VIP administration was found to reduce IOP by modulating F-actin distribution and the F-/G-actin ratio, which is closely related to cell stiffness [23]. It has been reported that high glucose (HG) + IL-1*β*-induced reduction of ZO-1 in ARPE19 cells was reversed by VIP [24]. Thus, we aimed to investigate whether VIP regulates the cytoskeleton through ZO-1.

In this study, we revealed that chronic ocular hypertension (COH) elicited junction disruption and ZO-1 reduction in the inner wall of the SC. The essential role of ZO-1 in maintaining the F/G-actin ratio and F-actin distribution was revealed by the knockdown of ZO-1 in 293 cells. We also demonstrated that VIP treatment attenuated ZO-1 decrease induced by COH in vivo or H_2_O_2_ in vitro. VIP exerts neuroprotective effects by inhibiting caspase-3 activation, which mediates ZO-1 reduction [25–27]. We further revealed that caspase-3 activation was decreased in the VIP+ H_2_O_2_ group, which may have promoted ZO-1 degradation through the endolysosomal pathway.

In contrast with IOP-lowering drugs targeting cell stiffness in the TM-SC pathway, which tends to elicit the disruption of ZO-1, VIP reduces cell stiffness under the premise of stabilizing cell-cell junctions [28]. This study increased our understanding of the regulatory role of VIP in stabilizing the interaction between the actin cytoskeleton and cell junctions and suggests a promising target strategy for glaucoma treatment.

## 2. Methods

### 2.1. Animals

Male Sprague-Dawley (SD) rats (6 weeks old), C57BL/6 mice (8 weeks), *Tie2Cre^/+^* mice, and *AnxA1^flox/flox^* mice were purchased from Gempharmatech (Nanjing, Jiangsu, China). Animals were fed with standard food and water in a 12 h light/dark cycle. All the animal protocols and procedures were in accordance with the Association for Research in Vision and Ophthalmology (ARVO) Statement for the Use of Animals in Ophthalmic and Vision Research and the Use Committee of Huazhong University of Science and Technology.

### 2.2. Reagents, Plasmid Construction, siRNA, and Antibodies

VIP (HSDAVFTDNYTRLRKQMAVKKYLNSILN, ≥98%) was synthesized by Sangon Biotech (Shanghai, China). Staurosporine (569397) and Ac-DEVD-CHO (235420) were purchased from Sigma-Aldrich. Full length human ZO-1 or caspase-3 was cloned into pcDNA3.1 (+). Sequence (5′ to 3′) of *siZO-1*: *si-1*: GUUAUACGAGCGAUCUCAU, *si-2*: GGAGGAAACAGCUAUAUGG. Sequence (5′ to 3′) of *sicaspase-3: si-1:* CCGACAAGCUUGAAUUUAU, *si-2*: GAAUUGAUGCGUGAUGUUU. The shRNA sequence that targets mouse ANXA1 sequence was designed as follows: 5′-GCCTCACAACCATCGTGAAGT-3′; adenovirus vectors expressing shANXA1 were constructed and generated by BrainVTA (Wuhan, China). The antibodies used in this study were as follows: anti–ZO-1 (Invitrogen, 33-9100), anti-cleaved caspase-3 (CST, 9661S), anti-caspase 3 (Proteintech, 19677-1-AP), anti-FLAG (CST, 14793S), anti-FLAG (Proteintech, 66008-3-Ig), anti-LAMP1 (CST, 15665S), anti-GAPDH (Proteintech, 60004-1-Ig), anti-*β*-actin (Proteintech, 60008-1-Ig), F-actin Staining Kit (Abcam, ab112127 and ab112125), and Fluorescein isothiocyanate-dextran (Sigma, FD70S).

### 2.3. Cell Culture and Transfection

Human embryonic kidney 293 cell line (HEK293) was purchased from China Center for Type Culture Collection (CCTCC). Human umbilical vein endothelial cells (HUVECs) were given as a gift from the division of Cardiology, Tongji Hospital. HEK293 and HUVECs were cultured in DMEM (GIBCO, Gaithersburg, MD, USA) supplemented with 10% fetal bovine serum (GIBCO) and 1% penicillin/streptomycin (Invitrogen) at 37°C with 5% CO2.

For transfection experiments, cells were seeded in 6-well or 24-well plates. At 60-70% confluence, cells were transfected with plasmid DNA or siRNA using Lipofectamine 3000 (Invitrogen). Cells were harvested for further analyses 48 h after transfection.

### 2.4. Immunofluorescence

Cells grown on glass coverslips were fixed in 4% paraformaldehyde for 15 min at room temperature. After being washed twice with PBS, cells were permeabilized with 0.1% Triton X-100 for 10 min and blocked with 5% BSA for 1 h at room temperature. These cells were then incubated with anti–ZO-1 (Invitrogen, 1 : 100), anti-FLAG (CST, 1 : 500), or anti-LAMP1 (CST, 1 : 100) at 4°C overnight, followed by appropriate secondary antibodies. For tissues, enucleated eyes were fixed in 4% paraformaldehyde for 2 h at room temperature, embedded in paraffin, and sectioned into 4 *μ*m. Frozen eyes were prepared and sectioned into 10 *μ*m. Paraffin sections and frozen sections for immunofluorescence were prepared using the same protocol as for the cells (see above). Images were captured using an inverted confocal microscope (Olympus FV3000). Three random visual fields were analyzed, and the average was taken for each group in cells. Six sections were analyzed, and the average was taken for each tissue in SD rats or mice.

### 2.5. TEM Imaging

Enucleated eyes were fixed in 2.5% glutaraldehyde at 4°C overnight, then washed three times with PBS, and fixed again in 2% osmium tetroxide for 2 h at room temperature. Following dehydrated in serial dilutions of ethanol, enucleated eyes were embedded in Epon. Thin sections (80 nm) of eyes were obtained using an Ultracut microtome (Leica), stained with 2% uranyl acetate for 15 min at room temperature, and then dried overnight. Prepared sections were observed and photographed by TEM (FEI Tecnai G2 20 TWIN, USA).

### 2.6. Western Blotting

Cell lysates were obtained in RIPA Buffer (Beyotime, P0013) containing protease and phosphatase inhibitor cocktails, and protein concentrations were quantified using BCA Protein Assay Kit (Beyotime, P0012). Subsequently, cell lysates were boiled in loading buffer for 5 min and subjected to SDS–PAGE, transferred to polyvinylidene fluoride (PVDF) membranes (Millipore). Membranes were then blocked with 5% nonfat milk in Tris-buffered saline/Tween 20 (TBST) at room temperature for 1 h and incubated with anti–ZO-1(Invitrogen, 1 : 500), anti-FLAG (Proteintech, 1 : 1000), anti-cleaved caspase-3 (CST, 1 : 1000), anti-caspase 3 (Proteintech, 1 : 1000), anti-GAPDH (Proteintech, 1 : 20000), and anti-*β*-actin (Proteintech, 1; 20000) at 4°C overnight. Following incubation with the corresponding HRP-conjugated antibody (Proteintech, 1 : 100000). Chemiluminescence signal were detected using the WesternBright ECL (Advansta) according to the manufacturer's instructions.

### 2.7. Coimmunoprecipitation

For coimmunoprecipitation (Co-IP) assays, freshly extracted cell lysates were incubated with 5 *μ*l anti–ZO-1 for 1 h at 4° C. Subsequently, add 20 *μ*l of resuspended Protein A/G PLUS-Agarose, incubated at 4° C on a rotary shaker overnight. The agarose beads were centrifuged (2500 rpm, 5 min), discard supernatant, and washed 4 times with 1.0 ml RIPA buffer. The precipitates were resuspended in 40 *μ*l SDS loading buffer and boiled for 5 min, further analyzed by western blot analysis according to standard procedures.

### 2.8. Establishment of Chronic Ocular Hypertension Model

Chronic ocular hypertension model in rats was induced by episcleral vein cauterization (EVC) as previously described. Briefly, rats were anesthetized with intraperitoneal injection of ketamine (60 mg/kg) and xylazine (5 mg/kg). Following application of topical anesthetic (proparacaine, 0.5% wt/vol eye drop), limbal periphery incisions were made on conjunctiva and Tenon's capsule. Three of the episcleral veins in right eye were identified, cauterized with an ophthalmic cautery. Sham surgery (without cauterization) was performed on the left eye. The incisions were carefully sutured, and levofloxacin eye drops (0.5%) were topically applied tid to prevent infection. Both eyes received drug interventions through eye dropping three times a day (9 : 00 AM/3 : 00 PM/9 : 00 PM) during day 14 to 27 postcauterization.

### 2.9. Paracellular Permeability to Fluorescein Isothiocyanate (FITC) Dextran

In vitro, paracellular permeability is evaluated using 70 kDa fluorescein isothiocyanate (FITC) dextran. HUVEC were seeded on the top transwell chamber with 0.4 *μ*m pore-size membrane (Corning, 3413) and grown for a minimum of 2 days until full confluence. Cells were treated with H_2_O_2_ (200 *μ*M) with or without VIP (50 *μ*M) for 6 h at 37°C, followed by 3 washes with PBS. FITC-dextran of 70 kDa (Sigma) was added to the top chamber of the Transwell to a final concentration of 1 mg/mL. After 1.5 hours, the sample was collected from the bottom chamber and read in a fluorescence microplate reader (Synergy2, BioTek, Winooski, VT, USA) at 485/528 nm.

In vivo, rats were anesthetized with intraperitoneal injection of ketamine and xylazine. For anterior chamber injection, a puncture was made using a 30G needle. Subsequently, a 33G a microsyringe (Hamilton) was then used to inject 5 *μ*l of 70 kDa FITC-dextran (1 mg/ml) into the anterior chamber. Samples were collected at indicated time points.

### 2.10. Statistical Analyses

All data are presented as the means ± SD from at least three independent experiments. The statistical analyses were performed using the software GraphPad Prism software (version 1.5.2, GraphPad Software Inc.). Comparisons among multiple groups were assessed using one-way analysis of variance (ANOVA) test, as indicated in the figure legends. Comparisons among two groups were assessed using Student's *t* test. A value of *P* < 0.05 was considered statistically significant.

## 3. Results

### 3.1. Persistent Elevated IOP Elicits Junction Disruption in the Inner Wall of Schlemm's Canal (SC)

**Figure 1:**
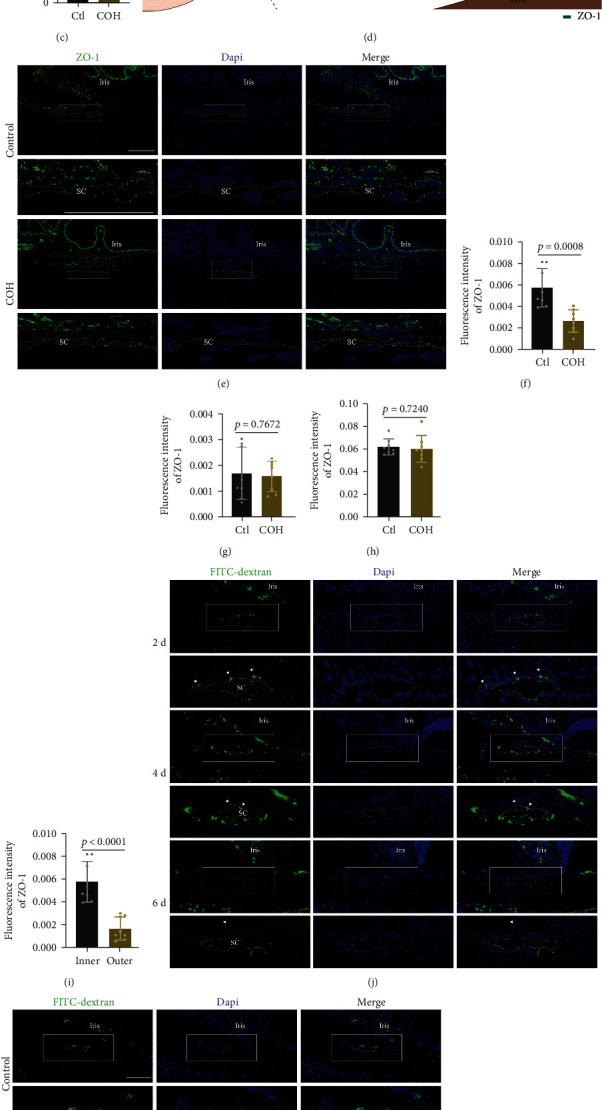
The expression pattern of ZO-1 in the inner wall of Schlemm's canal (SC). (a) Representative transmission electron microscopy (TEM) images of junctions in the SC inner wall of COH rats. The bottom row shows enlarged images of red boxes in the row above. Bar = 1 *μ*m. (b) and (c) Quantitative analysis (*t* test) of intercellular width and overlapping length in the SC inner wall. *n* = 8. (d) Schematic of the AH outflow pathway (left) and the expression pattern of ZO-1 in SC (right). (e) Representative images of ZO-1 immunofluorescence staining (green) in SC. Rows 2 and 4 show enlarged images of white boxes in rows 1 and 3. Dashed lines outline SC. Bar = 50 *μ*m. Quantitative analysis (*t* test) of ZO-1 fluorescence intensity in inner (f) and outer wall (g) of SC in COH rats. *n* = 8. (h) Quantitative analysis (*t* test) of ZO-1 fluorescence intensity in the TM of COH rats. *n* = 8. (i) Quantitative analysis (*t* test) of ZO-1 fluorescence intensity in the SC of untreated rats. *n* = 8. (j) and (l) Representative images and quantitative analysis (ANOVA) of residual FITC-dextran (green) in the TM-SC region of untreated rats 2, 4, 6 d after injection. Rows 2, 4, and 6 show enlarged images of white boxes in rows 1, 3, and 5. Dashed lines outline SC. White arrows indicate FITC-dextran. *n* = 8. Bar = 50 *μ*m. (k) and (m) Representative images and quantitative analysis (*t* test) of residual FITC-dextran (green) in the TM-SC region of COH rats 4 d after injection. Rows 2 and 4 show enlarged images of white boxes in rows 1 and 3. Dashed lines outline SC. *n* = 8. Bar = 50 *μ*m. Data are presented as the mean ± SD. COH: chronic ocular hypertension; SC: Schlemm's canal; CM: ciliary body; TM: trabecular meshwork.

At the outset, transmission electron microscopy (TEM) was performed to investigate the ultrastructure of junctions between the endothelial cells of Schlemm's canal (SCECs) (Figure 1(a)). Junctions with high electron density and overlaps were observed between adjacent SCECs, which are consistent with previous reports [16, 17]. The intercellular width of SCECs in chronic ocular hypertension (COH) rat models was markedly increased from 19.63 to 74.07 nm, while no difference was observed in the overlapping length (Figures 1(b) and 1(c)). Immunofluorescence staining of the anterior segment revealed that ZO-1 was expressed in the TM and SC tissues (Figures 1(d) and 1(e)). The levels of ZO1 in the inner wall of the SC, neither in the outer wall of the SC nor TM region, were significantly decreased in response to COH (Figures 1(f)–1(h)). We also found that ZO-1 in the outer wall of the SC was less than that in the inner wall (Figure 1(i)). The inner wall of the SC plays a dual role in maintaining AH homeostasis. It acts as a filter to allow AH to drain from the anterior chamber and simultaneously contributes to the blood-aqueous barrier because it is composed of a continuous endothelium [29]. Thus, we evaluated the permeability of the SC inner wall to a macromolecule (FITC-dextran, 70 kDa) in the TM-SC pathway. After intracameral injection of a large-molecule tracer, the fluorescence intensity of the TM-SC region diminished over time and reached a low level at 6 days in normal rats but 4 days in COH rats (Figures 1(j)–1(m)). These results indicate that persistently elevated IOP elicits junction disruption and a decrease in ZO-1 in the inner wall of the SC.

### 3.2. Knockdown of ZO-1 Induces Increases in the F/G-Actin Ratio and F-Actin Redistribution

**Figure 2:**
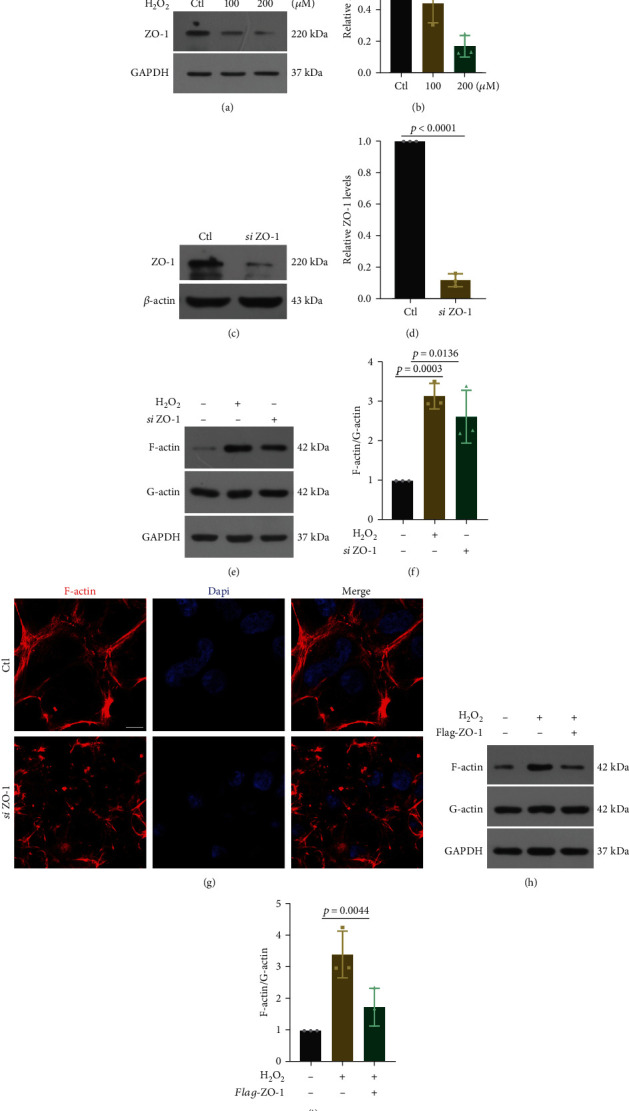
The function of ZO-1 on F/G-actin ratio and F-actin redistribution. (a) and (b) HEK 293 cells were treated with H_2_O_2_ (0, 100, and 200 *μ*M) for 6 h. Representative western blot images and quantitative analysis (*t* test) of ZO-1 levels. *n* = 3. (c) and (d) Representative western blot images and quantitative analysis (*t* test) of ZO-1 levels in HEK 293 cells transfected with *siZO-1*, *n* = 3. (e) and (f) Representative western blot images and quantitative analysis (*t* test) of F/G-actin ratio after H_2_O_2_ treatment (200 *μ*M, 6 h) or ZO-1 knockdown. *n* = 3. (g) Representative image of phalloidin stained F-actin (red) in HEK 293 cells transfected with *siZO-1*. Bar = 5 *μ*m. (h) and (i) Representative western blot images and quantitative analysis (ANOVA) of F/G-actin ratio after H_2_O_2_ treatment (200 *μ*M, 6 h) in HEK 293 cells transfected with ZO-1 overexpression plasmid. *n* = 3. Data are presented as the mean ± SD.

Previous research has revealed that the F-actin distribution in the SC became disordered in COH rats [22]. To investigate the impact of reduced ZO-1 on the cytoskeleton, we used H_2_O_2_ to induce oxidative stress injury in HEK 293 cells. As expected, the ZO-1 levels demonstrated a concentration-dependent response to H_2_O_2_ treatment (Figures 2(a) and 2(b)). However, H_2_O_2_ showed a rather complicated regulation of actin dynamics and distribution; hence, we also transfected 293 cells with small interfering RNA against ZO-1 (*siZO-1*). The interference efficiency was determined by western blotting (Figures 2(c) and 2(d)). After H_2_O_2_ treatment or ZO-1 knockdown, the F/G-actin ratio in 293 cells markedly increased (Figures 2(e) and 2(f)). In the control group, F-actin was strongly distributed within the vicinity of the plasma membrane. In the ZO-1 knockdown group, F-actin showed a disorganized pattern (Figure 2(g)). Furthermore, ZO-1 overexpression attenuated the increase in the F/G-actin ratio induced by H_2_O_2_ (Figures 2(h) and 2(i)). These in vitro data indicate the essential role of ZO-1 in maintaining the organization of the cytoskeleton.

### 3.3. VIP Attenuates Junction Disassembly and a Decrease in ZO-1

**Figure 3:**
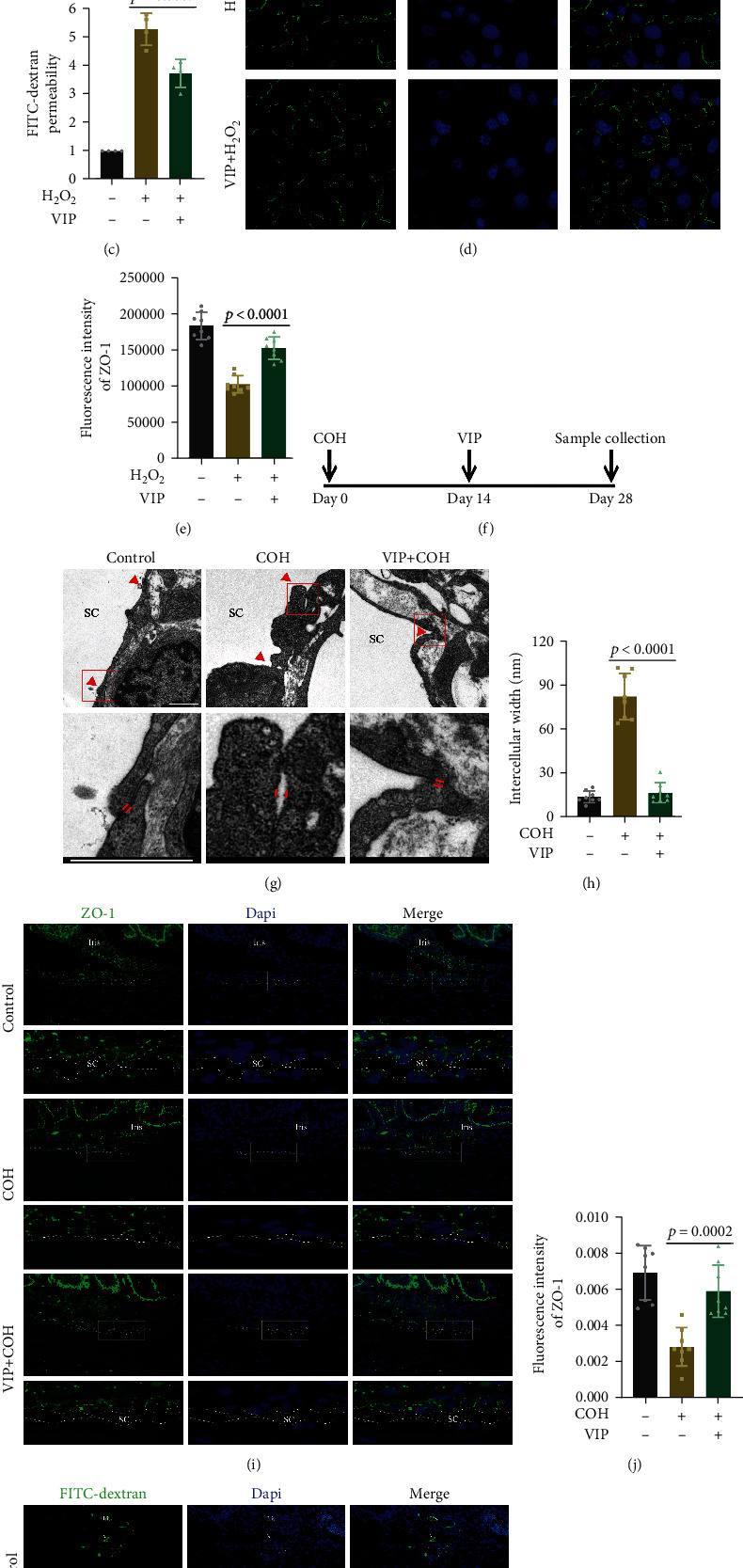
The function of VIP treatment on junction disassembly and ZO-1 decrease. (a) and (b) Representative western blot images and quantitative analysis (ANOVA) of ZO-1 levels in H_2_O_2_ (200 *μ*M, 6 h)-stimulated HUVECs pretreated with VIP (0, 10, and 50 *μ*M). *n* = 3. (c) Quantitative analysis (ANOVA) of FITC-dextran permeability in monolayer of HUVECs pretreated with VIP (50 *μ*M). *n* = 3. (d) and (e) Representative images and quantitative analysis (ANOVA) of ZO-1 immunofluorescence staining (green) in HUVECs pretreated with VIP (50 *μ*M). *n* = 8. Bar = 30 *μ*m. (f) The timeline for VIP treatment. VIP (50 *μ*M) was topically administrated in COH rats for 2 weeks before sample collection. (g) Representative TEM images of junctions between SCECs. The bottom row shows enlarged images of red boxes in the row above. Red arrows indicate intercellular region. Bar = 1 *μ*m. (h) Quantitative analysis (ANOVA) of intercellular width in SCECs. *n* = 8. (i) and (j) Representative images and quantitative analysis (ANOVA) of ZO-1 immunofluorescence staining (green) in SCECs. Rows 2, 4, and 6 show enlarged images of white boxes in rows 1, 3, and 5. Dashed lines outline SC. *n* = 8. Bar = 50 *μ*m. (k) and (l) Representative images and quantitative analysis (ANOVA) of residual FITC-dextran (green) in the TM-SC region. Rows 2, 4, and 6 show enlarged images of white boxes in rows 1, 3, and 5. Dashed lines outline SC. White arrows indicate FITC-dextran. *n* = 8. Bar = 50 *μ*m. Data are presented as the mean ± SD. COH: chronic ocular hypertension; SC: Schlemm's canal.

To determine whether VIP influences junction stability and ZO-1 levels, VIP gradients were measured in H_2_O_2_-treated human umbilical vein endothelial cells (HUVECs). Pretreatment with VIP increased the ZO-1 expression in a concentration-dependent manner; thus, 50 *μ*M was applied as follows (Figures 3(a) and 3(b)). ZO-1 is normally located in the peripheral cytoplasm along the membrane, which is similar to previous reports [30, 31]. After H_2_O_2_ treatment, the distribution of ZO-1 showed obvious discontinuity and it was absent in some intercellular spaces. VIP increased the ZO-1 levels and improved the intercellular distribution (Figures 3(c) and 3(d)). To determine whether the junctional function was restored, we performed a FITC-dextran permeability assay using a transwell. HUVECs were grown to confluence on transwell membranes, and H_2_O_2_ was added with or without VIP. We found that VIP diminished the H_2_O_2_-induced high permeability of the HUVEC monolayers to 70 kDa dextran (Figure 3(e)). VIP was topically administered to COH rats for 2 weeks (Figure 3(f)). Electron microscopy analysis revealed that the intercellular width of the SCECs significantly decreased in the VIP-treated group compared with the COH group (Figures 3(g) and 3(h)). Moreover, we observed increased ZO-1 levels in the SC inner wall as a result of VIP administration (Figures 3(i) and 3(j)). Correspondingly, VIP promoted the normalization of residual FITC-dextran in the TM-SC region 3 days after injection (Figures 3(k) and 3(l)). These results indicate that VIP prevents junction disassembly and a decrease in ZO-1, as well as blood-aqueous barrier disruption.

### 3.4. VIP Rescues ZO-1 Levels and Distribution through Inhibiting Caspase-3

**Figure 4:**
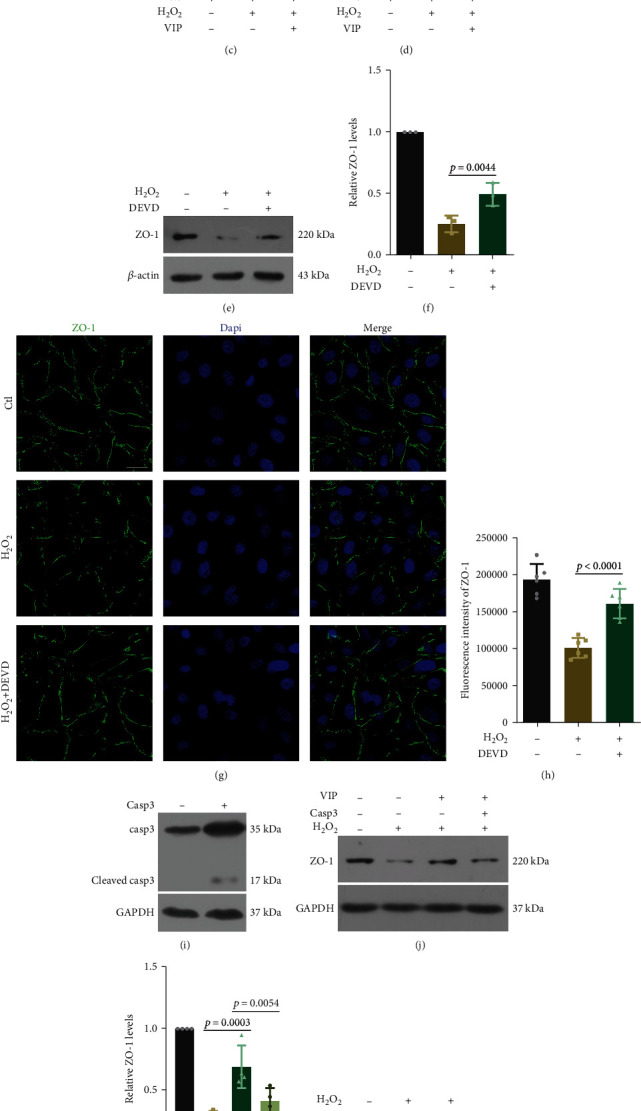
VIP regulates ZO-1 expression via caspase-3. (a)–(c) HUVECs were pretreated with VIP (50 *μ*M) for 2 h before H_2_O_2_ (200 *μ*M, 6 h). Representative western blot images and quantitative analysis (ANOVA) of cleaved caspase-3 and VPAC2 levels. *n* = 3. (d) Quantitative analysis (ANOVA) of caspase-3 activity using caspase-3 activity assay kit. *n* = 5. (e) and (f) HUVECs were pretreated with caspase-3 inhibitor (Ac-DEVD-CHO, 20 *μ*M) 30 min before H_2_O_2_ (200 *μ*M, 6 h). Representative western blot images and quantitative analysis (ANOVA) of ZO-1 levels in HUVECs. *n* = 3. (g) and (h) Representative immunofluorescence staining and quantitative analysis (ANOVA) of ZO-1 in HUVECs pretreated with caspase-3 inhibitor. *n* = 6. Bar = 30 *μ*m. (i) Representative western blot images of cleaved caspase-3 after overexpression of full-length caspase-3. (j) and (k) Representative western blot images and quantitative analysis (ANOVA) of ZO-1 levels in HEK 293 cells transfected with caspase-3 plasmid in the presence of H_2_O_2_ (200 *μ*M) and VIP (50 *μ*M). *n* = 4. (l) and (m) Representative western blot images and quantitative analysis (ANOVA) of ZO-1 levels HEK 293 cells transfected with *siCasp3* in the presence of H_2_O_2_ (200 *μ*M). *n* = 3. (n) Coimmunoprecipitation of ZO-1 and cleaved caspase-3. Data are presented as the mean ± SD.

A previous study revealed that VPAC2 was the main receptor for VIP in the SCE of SD rats [22]. VPAC2 serves as a receptor for both VIP and PACAP. Agonists for VPAC2 (for example, VIP, PACAP, and PHI) have been proven to exert neuroprotective effects by inhibiting caspase-3 activation in astrocytes and neurons [25, 32, 33]. Since caspase-3 mediates the reduction of ZO-1, we investigated whether VIP rescues ZO-1 levels and distribution via caspase-3 inhibition [26]. It was revealed that VIP reduced cleaved caspase-3 levels and caspase-3 activity in H_2_O_2_-treated HUVECs, while no change was observed in VPAC2 expression (Figures 4(a)–4(c)). The caspase-3 specific inhibitor, Ac-DEVD-CHO, increased the ZO-1 levels and distribution along the membrane, which was consistent with VIP (Figures 4(d)–4(h)). Knockdown of caspase-3 by si-cas3 also rescued the ZO-1 levels (Figures 4(i) and 4(j)). To further confirm the role of caspase-3, we overexpressed caspase-3 in the presence of VIP. Overexpression of full-length caspase-3 elicited an increase in the cleaved band at 17 kDa and reversed the upregulation effect of VIP on ZO-1 (Figures 4(k)–4(m)). We immunoprecipitated ZO-1 and blotted cleaved caspase-3 to detect the binding between these two proteins. It was demonstrated that cleaved caspase-3 interacted with ZO-1 in response to H_2_O_2_, and this interaction was diminished when VIP was administered (Figure 4(n)). A previous study reported that ZO-1 was fragmented into cleavage products in apoptotic H184A1 cells [27]. We generated a full-length ZO-1 with a C-terminal FLAG tag; unfortunately, no fragment was detected in H_2_O_2_-treated HUVECs, suggesting cleavage-independent mechanisms by which caspase-3 activation causes ZO-1 reduction.

### 3.5. Caspase-3 Promotes ZO-1 Degradation through Endolysosomal Pathway

**Figure 5:**
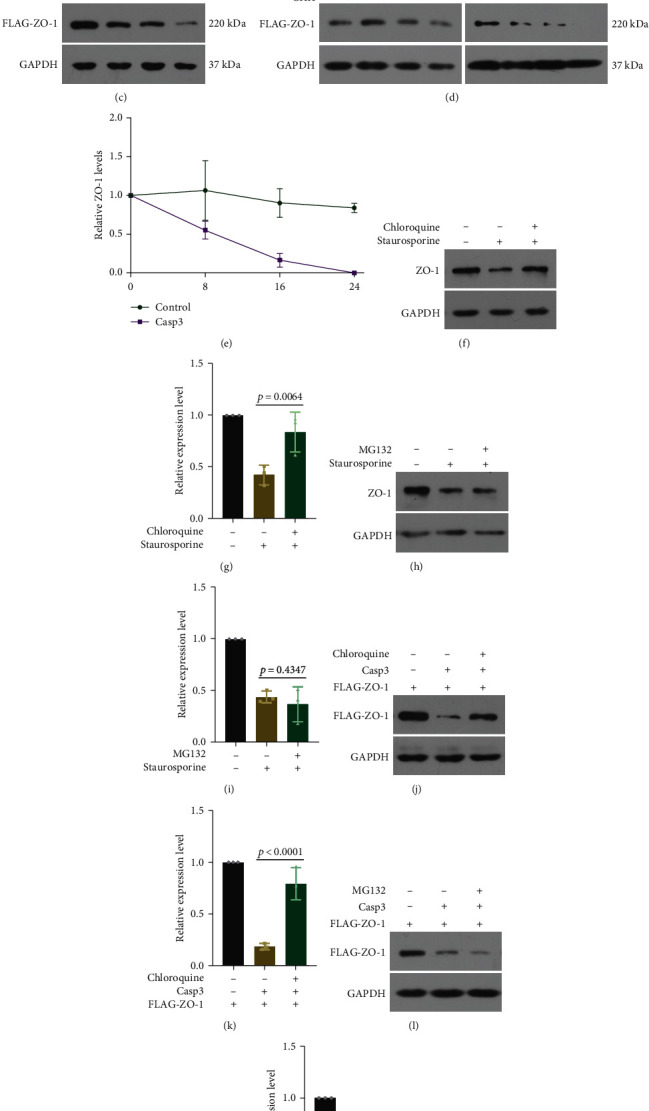
Caspase-3 promotes ZO-1 degradation. (a) RT-PCR analysis (*t* test) of ZO-1 mRNA levels in the HEK 293 cells transfected with caspase-3 overexpression plasmid. *n* = 4. Endogenous (b) and exogenous (c) ZO-1 were detected by western blot in HEK 293 cells transfected with the caspase-3 expression plasmid gradient. (d) and (e) At 48 h after caspase-3 transfection, CHX was added and incubated for indicated time to evaluate the half-life of exogenous ZO-1 protein in HEK 293 cells. *n* = 3. (f)–(i) Chloroquine (30 *μ*M) or MG-132 (10 *μ*M) was added 2 h before staurosporine treatment (20 nM, 3 h). Representative western blot images and quantitative analysis (ANOVA) of endogenous ZO-1 levels in HEK 293 cells. *n* = 3. (j)–(m) Chloroquine (30 *μ*M) and MG-132 (10 *μ*M) were added in HEK 293 cells transfected with the caspase-3 overexpression plasmid. Representative western blot images and quantitative analysis (ANOVA) of exogenous ZO-1 levels. *n* = 3. Data are presented as the mean ± SD.

**Figure 6:**
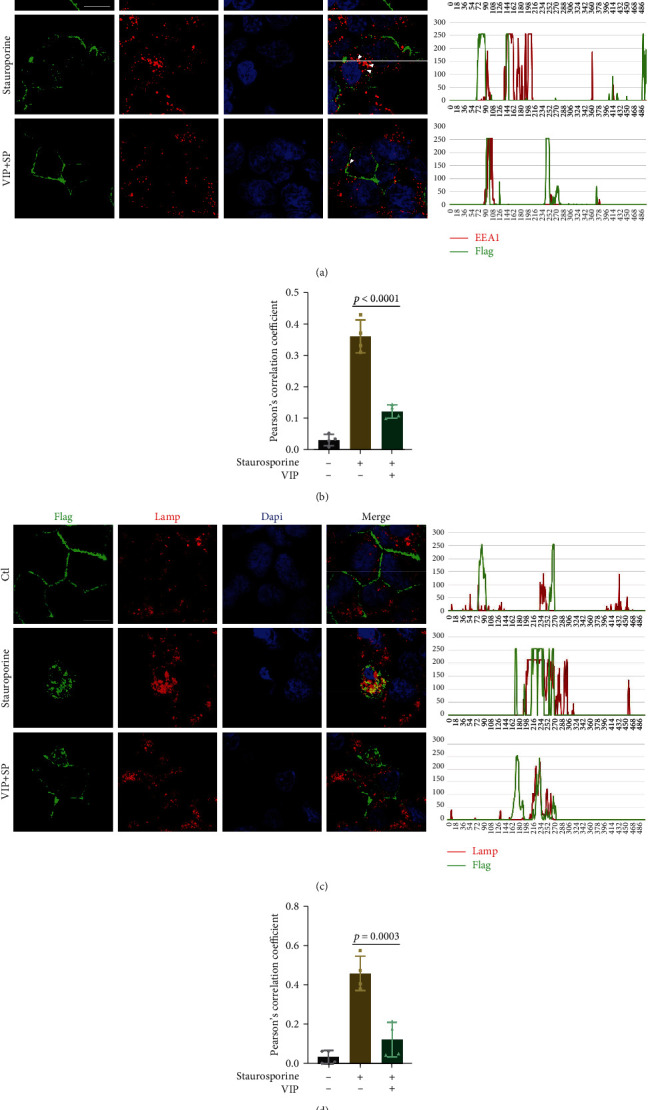
Endo-lysosomal pathway determines ZO-1 degradation. HEK 293 cells were treated with staurosporine (20 nM) and VIP (50 *μ*M) for 6 h. (a) and (b) Representative images and quantitative analysis (ANOVA) of colocalization (white arrows) between FLAG-ZO-1 and EEA1. *n* = 4. (c) and (d) Representative images and quantitative analysis (ANOVA) of colocalization between FLAG-ZO-1 and LAMP1. *n* = 4. Bar = 20 *μ*m. Data are presented as the mean ± SD.

Overexpression of caspase-3 had no impact on ZO-1 mRNA levels; thus, we focused on the degradation process (Figure 5(a)). The gradients of the caspase-3 plasmid were transfected into 293 cells, and decrements in endogenous ZO-1 and exogenous FLAG-ZO-1 were observed (Figures 5(b) and 5(c)). A time-course evaluation using 10 *μ*g/ml of cycloheximide (CHX) revealed that ZO-1 was undetectable 24 h after CHX treatment in the presence of caspase-3 overexpression, indicating a shorter half-life than normal (Figures 5(d) and 5(e)). Lysosomes (chloroquine) and ubiquitin-proteasome (MG-132) inhibitors were used to determine the contribution of two major protein degradation pathways. We used two approaches to increase caspase-3 activity: staurosporine for endogenous ZO-1 and overexpression plasmid of caspase-3 for exogenous ZO-1. ZO-1 reduction induced by staurosporine was blocked with Ac-DEVD-CHO, suggesting a vital role for caspase-3 in this process. Chloroquine reversed ZO-1 reduction induced by the two stimuli, while MG-132 showed no effect, implying that caspase-3 may downregulate ZO-1 via the lysosomal degradation pathway (Figures 5(f)–5(m)). Consistent with the quantification results for protein levels, ZO-1 was colocalized with the early endosome marker EEA1 and lysosome marker LAMP1 6 h after staurosporine stimulation, suggesting that the endolysosomal system plays a vital role. VIP treatment reduced this colocalization (Figures 6(a)–6(d)). These results indicate that caspase-3 activation contributes to the lysosomal degradation of ZO-1.

### 3.6. The Effect of VIP in Modulating Cytoskeleton

**Figure 7:**
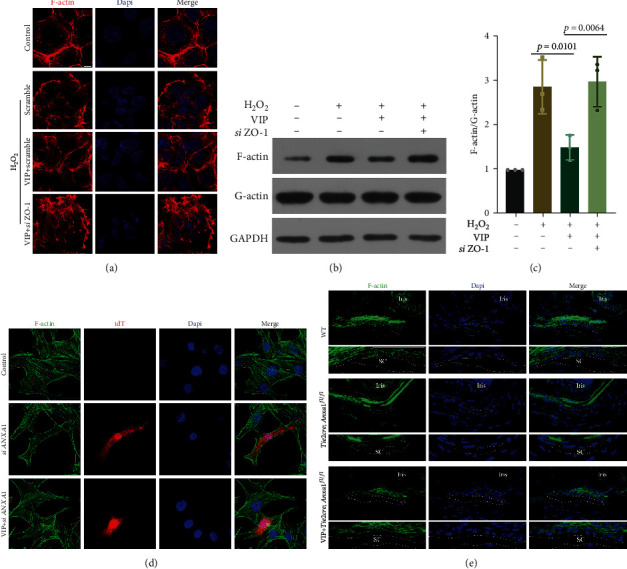
The effect of VIP on ZO-1 dependent and nondependent pathway in cytoskeleton modulation. VIP (50 *μ*M) was administrated 2 h before H_2_O_2_ (200 *μ*M) in HEK 293 cells transfected with *siZO-1*. (a) Representative images of phalloidin stained F-actin (red). Bar = 5 *μ*m. (b) and (c) Representative western blot images and quantitative analysis (ANOVA) of F/G-actin ratio. *n* = 3. (d) Representative images of phalloidin stained F-actin (green) in AnxA1 knockdown HUVECs. Bar = 30 *μ*m. (e) Representative image of phalloidin stained F-actin (green) in SC of *Tie2Cre/+*, *AnxA1^flox/flox^* mice. Rows 2, 4, and 6 show enlarged images of white boxes in rows 1, 3, and 5. Dashed lines outline SC. Bar = 40 *μ*m. Data are presented as the mean ± SD.

To verify whether VIP modulates the cytoskeleton through junction stabilization in H_2_O_2_-treated 293 cells, *siZO-1* was applied to the VIP group. VIP reduced the disorganization of F-actin and the elevation of the F/G-actin ratio in response to H_2_O_2_; however, ZO-1 knockdown blocked this effect (Figures 7(a)–7(c)). A previous study revealed *that AnxA1−/−* mice demonstrated a distorted actin cytoskeleton accompanied by ZO-1 disruption in brain microvascular endothelial cells [34]. Thinner F-actin fibrils were observed in AnxA1 knockdown HUVECs, and VIP treatment induced the appearance of clear transcellular F-actin fibrils, suggesting a more normal actin organization (Figure 7(d)). SC is characterized as a lymphatic-like vessel that expresses endothelial and lymphatic valve proteins such as Tie2 and FOXC2 [35, 36]. We employed *Tie2Cre^/+^*, *AnxA1^flox/flox^* (TG) mice, which showed spontaneous F-actin redistribution in Schlemm's canal. Consistent with the in vitro results, VIP administration attenuated this redistribution (Figure 7(e)). However, further studies are required to clarify whether VIP can also regulate cytoskeleton through ZO-1-dependent mechanisms in TG mice. These results demonstrated the role of VIP in modulating F-actin distribution and dynamics.

## 4. Discussion

**Figure 8:**
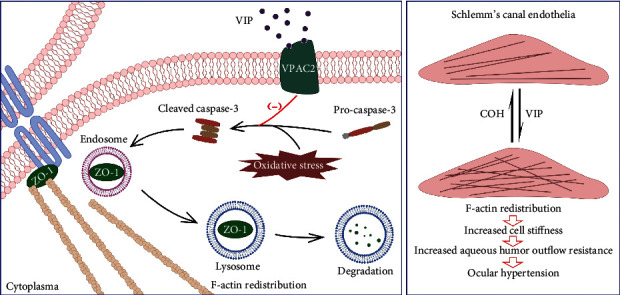
A proposed mechanism by which VIP regulates cytoskeleton. Oxidative stress-induced caspase-3 activation elicits ZO-1 degradation through endolysosomal pathway, causes F-actin redistribution, further results in cell stiffness increase and AH outflow resistance. VIP treatment reduces ZO-1 endolysosomal degradation by inhibiting capasase-3, thus, plays a protective role in cytoskeleton.

The exact pathogenesis of IOP elevation in patients with POAG remains unclear. Unlike the sinusoidal endothelium (discontinuous and without a basement membrane), the SC consists of a continuous endothelium with a discontinuous basement membrane. The inner wall of the SC served as the final filtration barrier of the AH outflow. The increased cell stiffness of SCE may contribute to increased resistance of the AH outflow pathway. Previous research has described the disordered F-actin distribution of SC in COH rats, and VIP treatment stabilizes the actin cytoskeleton via the Sp1–LRRK2 pathway [22, 37]. We demonstrated that junction disruption and ZO-1 reduction are prominent causes of abnormal F-actin distribution and actin polymerization. VIP treatment attenuates ZO-1 lysosomal degradation through caspase-3 inhibition, thus, promoting F-actin to a normal distribution (Figure 8).

Despite the relatively low flow rate of AH, the shear stress in SC is estimated to range from 2 to 20 dynes/cm^2^ at an elevated IOP, which is comparable to that in large arteries (2–25 dynes/cm^2^) [38, 39]. Elevated shear stress induces oxidative stress via different pathways, such as TLR4 activation and MAP kinase tyrosine phosphorylation [40–42]. Oxidative stress causes various forms of damage to the TM, such as ECM accumulation [43], DNA damage [44], cytoskeletal rearrangement, and cell loss [45], ultimately resulting in reduced outflow facilities and increased IOP. However, little is known about how oxidative stress causes SCE damage. Lei et al. reported that angular aqueous plexus (functional equivalent to human SC) endothelial cells from porcine eyes showed increased levels of junction proteins after exposure to 40% oxygen for 14 days in vitro [46]. In contrast, we revealed that persistently elevated IOP elicited junction disruption and ZO-1 reduction in the inner wall of the SC, suggesting a rather complex mechanism in vivo. In the present study, we surmise that the accumulated oxidative stress induced by increased shear stress on SCE led to ZO-1 reduction. The interaction between ZO-1 and actin may be involved in multiple biological processes, including cell polarity, junction assembly, barrier permeability, actin distribution, and actin dynamics [47]. The deficiency of ZO-1 led to myosin II activation, stress fiber formation, and loss of junctional mechanotransducers such as vinculin and PAK2 [48]. In our experiments, ZO-1 knockdown or oxidative damage elicited the redistribution of F-actin and the elevation of the F/G-actin ratio. Overexpression of ZO-1 attenuated the F/G-actin ratio elevation induced by H_2_O_2_, indicating that increased actin polymerization is mediated in part by ZO-1 reduction during oxidative damage. Thus, we speculated that persistently elevated IOP may trigger oxidative stress in SCE, causing ZO-1 reduction and further leading to disordered F-actin and F/G-actin dynamics, which in turn increased AH outflow resistance.

VIP protects the distribution and levels of junction proteins (for example, ZO-1, occludin, claudin-3, and claudin-4) against colitis by inhibiting MLCK or PKC*ε* [49, 50]. The present data demonstrated that VIP attenuates the decrements in ZO-1 of SCE in COH rats and H_2_O_2_-treated HUVECs. H_2_O_2_ leads to G*α*12/Src-mediated tyrosine phosphorylation or PKC*α*-mediated serine phosphorylation of ZO-1, which induces the dissociation of ZO-1 from junctions in epithelial and endothelial cells [18, 19]. Our results indicate that caspase-3 activation may also be involved in oxidative stress-induced ZO-1 reduction since the caspase-3 specific inhibitor improved this impairment. Caspase-3 activation was observed in oxidative damage induced by various stimuli, such as CoCl_2_, glutamate, and H_2_O_2_ [51–53]. PACAP, which shares receptors with VIP, and the VPAC2 agonist peptide histidine isoleucine (PHI), has been reported to promote the deactivation of caspase-3 mainly through the inhibition of PKC signaling pathways in neurons [33, 54]. Similarly, VIP attenuates caspase-3 activation by interacting with the VPAC2 receptor, thus, protecting lung alveolar L2 cells from cigarette smoke extract-induced oxidative damage [55]. Therefore, we investigated whether VIP inhibits caspase-3 activation in H_2_O_2_-treated HUVECs. Consistent with previous reports, VIP decreased H_2_O_2_-induced caspase-3 activity, as detected by the cleaved caspase-3 levels and caspase-3 activity assay. We also verified the interaction between cleaved caspase-3 and ZO-1 using Co-IP, which was diminished after VIP administration. It was concluded that VIP may protect cell junctions and ZO-1 through the inhibition of caspase-3 activation. However, more experiments are needed to determine if there is an inhibitory effect of VIP on the catalytic activity of caspase-3 in addition to reducing cleaved caspase-3 levels, further unraveling the underlying mechanisms.

It has previously been shown that caspase-3 may contribute to ZO-1 reduction by cleaving ZO-1 into cleavage products in apoptotic cells independent of the apoptotic stimulus type [27]. We generated a full-length ZO-1 with a C-terminal FLAG tag to detect multiple fragments of different molecular weights. However, no fragments were detected in the H_2_O_2_-treated cells. We surmise that this is because the cells were harvested at a rather late apoptotic stage (signed by floating cells) in a previous study, while the cells in our experiment adhered at the end of H_2_O_2_-treatment, indicating the activation of caspase-3 without cell death. Additional mechanisms may underlie the activated caspase-3 reduction of ZO-1 independent of cleavage. Since caspase-3 has no impact on ZO-1 at the transcriptional level, we determined the degradation process of ZO-1. Under normal conditions, ZO-1 has a half-life greater than 24 h, which is shortened to approximately 8 h by the overexpression of caspase-3 in 293 cells. In virus-mediated endothelial barrier disruption, the proteasome inhibitor, MG132, but not the lysosomal inhibitor, chloroquine, was reported to attenuate ZO-1 degradation [56, 57]. Enhanced autophagy was also associated with the redistribution and degradation of ZO-1 after OGD/R and I/R injury [58]. In our study, caspase-3 may downregulate ZO-1 through the endolysosomal degradation pathway identified by colocalization with endosome marker EEA1 and lysosome marker LAMP1 6 h after staurosporine stimulation, which was reduced by VIP treatment. Although the endolysosomal pathway was observed to mediate ZO-1 degradation, the data did not exclude the autophagy pathway because the lysosomal inhibitor chloroquine also inhibits autophagic degradation [59].

## 5. Conclusion

The present study revealed that VIP stabilizes the cytoskeleton of Schlemm's canal endothelia by reducing caspase-3-mediated ZO-1 lysosomal degradation. We demonstrated that COH elicited junction disruption and ZO-1 reduction in the inner wall of the Schlemm's canal, which may result in F-actin redistribution, further increasing AH outflow resistance. The inhibition of ZO-1 degradation led to cytoskeleton protection in F-actin distribution and the F/G-actin ratio. VIP treatment reduced ZO-1 lysosomal degradation by inhibiting caspase-3. Thus, our investigation of VIP for stabilizing the cytoskeleton against COH offers novel therapeutic perspectives to reduce AH outflow resistance by maintaining the dynamic interaction between the cytoskeleton and cell junctions.

## Data Availability

The datasets generated during and/or analyzed during the current study are available from the corresponding author on reasonable request.

## Abbreviations

POAG: Primary open-angle glaucoma
AH: Aqueous humor
VIP: Vasoactive intestinal peptide
COH: Chronic ocular hypertension
IOP: Intraocular pressure
SC: Schlemm's canal
TM: Trabecular meshwork
SCE: Schlemm's canal endothelia
MLC: Myosin light chain
HG: High glucose.

## References

[1] Flaxman S. R., Bourne R. R. A., Resnikoff S. (2017). Global causes of blindness and distance vision impairment 1990-2020: a systematic review and meta-analysis. *The Lancet Global Health*.

[2] Rosenthal J., Leske M. C. (1980). Open-angle glaucoma risk factors applied to clinical area. *Journal of the American Optometric Association*.

[3] Wang N., Chintala S. K., Fini M. E., Schuman J. S. (2001). Activation of a tissue-specific stress response in the aqueous outflow pathway of the eye defines the glaucoma disease phenotype. *Nature Medicine*.

[4] Vahabikashi A., Gelman A., Dong B. (2019). Increased stiffness and flow resistance of the inner wall of Schlemm's canal in glaucomatous human eyes. *Proceedings of the National Academy of Sciences of the United States of America*.

[5] Overby D. R., Zhou E. H., Vargas-Pinto R. (2014). Altered mechanobiology of Schlemm's canal endothelial cells in glaucoma. *Proceedings of the National Academy of Sciences of the United States of America*.

[6] Tanna A. P., Johnson M. (2018). Rho kinase inhibitors as a novel treatment for glaucoma and ocular hypertension. *Ophthalmology*.

[7] Stack T., Vincent M., Vahabikashi A. (2020). Targeted delivery of cell softening micelles to Schlemm's canal endothelial cells for treatment of glaucoma. *Small*.

[8] Stack T., Vahabikashi A., Johnson M., Scott E. (2018). Modulation of Schlemm's canal endothelial cell stiffness via latrunculin loaded block copolymer micelles. *Journal of Biomedical Materials Research Part A*.

[9] Chaudhuri O., Parekh S. H., Fletcher D. A. (2007). Reversible stress softening of actin networks. *Nature*.

[10] Satcher R. L., Dewey C. F. (1996). Theoretical estimates of mechanical properties of the endothelial cell cytoskeleton. *Biophysical Journal*.

[11] Shi Y., Zhang L., Pu H. (2016). Rapid endothelial cytoskeletal reorganization enables early blood-brain barrier disruption and long-term ischaemic reperfusion brain injury. *Nature Communications*.

[12] Madara J. L., Moore R., Carlson S. (1987). Alteration of intestinal tight junction structure and permeability by cytoskeletal contraction. *The American Journal of Physiology*.

[13] Odenwald M. A., Choi W., Kuo W. T. (2018). The scaffolding protein ZO-1 coordinates actomyosin and epithelial apical specializations *in vitro* and *in vivo*. *Journal of Biological Chemistry*.

[14] Schwayer C., Shamipour S., Pranjic-Ferscha K. (2019). Mechanosensation of tight junctions depends on ZO-1 phase separation and flow. *Cell*.

[15] Otani T., Nguyen T. P., Tokuda S. (2019). Claudins and JAM-A coordinately regulate tight junction formation and epithelial polarity. *Journal of Cell Biology*.

[16] Burridge K., Wittchen E. S. (2013). The tension mounts: stress fibers as force-generating mechanotransducers. *The Journal of Cell Biology*.

[17] Hartsock A., Nelson W. J. (2008). Adherens and tight junctions: structure, function and connections to the actin cytoskeleton. *Biochimica et Biophysica Acta*.

[18] Abdala-Valencia H., Kountz T. S., Marchese M. E., Cook-Mills J. M. (2018). VCAM-1 induces signals that stimulate ZO-1 serine phosphorylation and reduces ZO-1 localization at lung endothelial cell junctions. *Journal of Leukocyte Biology*.

[19] Yu W., Beaudry S., Negoro H. (2012). H2O2 activates G protein, *α* 12 to disrupt the junctional complex and enhance ischemia reperfusion injury. *Proceedings of the National Academy of Sciences of the United States of America*.

[20] Cunha-Reis D., Caulino-Rocha A. (2020). VIP modulation of hippocampal synaptic plasticity: a role for VIP receptors as therapeutic targets in cognitive decline and mesial temporal lobe epilepsy. *Frontiers in Cellular Neuroscience*.

[21] Ji P., Chen L., Gong J. (2018). Co-expression of vasoactive intestinal peptide and protein gene product 9.5 surrounding the lumen of human Schlemm's canal. *Experimental Eye Research*.

[22] Chen L., Li M., Luo Z. (2018). VIP regulates morphology and F-actin distribution of Schlemm's canal in a chronic intraocular pressure hypertension model via the VPAC2 receptor. *Investigative Ophthalmology & Visual Science*.

[23] Zhou N., Lee J. J., Stoll S., Ma B., Costa K. D., Qiu H. (2017). Rho kinase regulates aortic vascular smooth muscle cell stiffness via actin/SRF/myocardin in hypertension. *Cellular Physiology and Biochemistry*.

[24] Scuderi S., D’Amico A. G., Castorina A., Imbesi R., Carnazza M. L., D’Agata V. (2013). Ameliorative effect of PACAP and VIP against increased permeability in a model of outer blood retinal barrier dysfunction. *Peptides*.

[25] Dejda A., Sokołowska P., Nowak J. Z. (2005). Neuroprotective potential of three neuropeptides PACAP, VIP and PHI. *Pharmacological Reports*.

[26] Zehendner C. M., Librizzi L., de Curtis M., Kuhlmann C. R., Luhmann H. J. (2011). Caspase-3 contributes to ZO-1 and Cl-5 tight-junction disruption in rapid anoxic neurovascular unit damage. *PLoS One*.

[27] Bojarski C., Weiske J., Schöneberg T. (2004). The specific fates of tight junction proteins in apoptotic epithelial cells. *Journal of Cell Science*.

[28] Kaneko Y., Ohta M., Inoue T. (2016). Effects of K-115 (Ripasudil), a novel ROCK inhibitor, on trabecular meshwork and Schlemm's canal endothelial cells. *Scientific Reports*.

[29] Stamer W. D., Braakman S. T., Zhou E. H. (2015). Biomechanics of Schlemm's canal endothelium and intraocular pressure reduction. *Progress in Retinal and Eye Research*.

[30] Lin Y., Zhang C., Xiang P., Shen J., Sun W., Yu H. (2020). Exosomes derived from HeLa cells break down vascular integrity by triggering endoplasmic reticulum stress in endothelial cells. *Journal of Extracellular Vesicles*.

[31] Li J., Li Z., Jiang P. (2018). Circular RNA IARS (circ-IARS) secreted by pancreatic cancer cells and located within exosomes regulates endothelial monolayer permeability to promote tumor metastasis. *Journal of Experimental & Clinical Cancer Research*.

[32] Botia B., Seyer D., Ravni A. (2008). Peroxiredoxin 2 is involved in the neuroprotective effects of PACAP in cultured cerebellar granule neurons. *Journal of Molecular Neuroscience*.

[33] Goursaud S., Focant M. C., Berger J. V., Nizet Y., Maloteaux J. M., Hermans E. (2011). The VPAC2 agonist peptide histidine isoleucine (PHI) up-regulates glutamate transport in the corpus callosum of a rat model of amyotrophic lateral sclerosis (hSOD1G93A) by inhibiting caspase-3 mediated inactivation of GLT-1a. *FASEB Journal*.

[34] Cristante E., McArthur S., Mauro C. (2013). Identification of an essential endogenous regulator of blood-brain barrier integrity, and its pathological and therapeutic implications. *Proceedings of the National Academy of Sciences of the United States of America*.

[35] Perkumas K. M., Stamer W. D. (2012). Protein markers and differentiation in culture for Schlemm's canal endothelial cells. *Experimental Eye Research*.

[36] Park D. Y., Lee J., Park I. (2014). Lymphatic regulator PROX1 determines Schlemm's canal integrity and identity. *The Journal of Clinical Investigation*.

[37] Yan X., Li M., Luo Z., Zhao Y., Zhang H., Chen L. (2020). VIP induces changes in the F-/G-actin ratio of Schlemm's canal endothelium via LRRK2 transcriptional regulation. *Investigative Ophthalmology & Visual Science*.

[38] Ethier C. R., Read A. T., Chan D. (2004). Biomechanics of Schlemm's canal endothelial cells: influence on F-actin architecture. *Biophysical Journal*.

[39] Lei Y., Stamer W. D., Wu J., Sun X. (2014). Endothelial nitric oxide synthase-related mechanotransduction changes in aged porcine angular aqueous plexus cells. *Investigative Ophthalmology & Visual Science*.

[40] Wang Z., Zhang J., Li B. (2014). Resveratrol ameliorates low shear stress-induced oxidative stress by suppressing ERK/eNOS-Thr495 in endothelial cells. *Molecular Medicine Reports*.

[41] Wang Z., Wang F., Kong X., Gao X., Gu Y., Zhang J. (2019). Oscillatory shear stress induces oxidative stress via TLR4 activation in endothelial cells. *Mediators of Inflammation*.

[42] Yeh L. H., Park Y. J., Hansalia R. J. (1999). Shear-induced tyrosine phosphorylation in endothelial cells requires Rac1-dependent production of ROS. *American Journal of Physiology-Cell Physiology*.

[43] Luna C., Li G., Qiu J., Epstein D. L., Gonzalez P. (2009). Role of miR-29b on the regulation of the extracellular matrix in human trabecular meshwork cells under chronic oxidative stress. *Molecular Vision*.

[44] Pulliero A., Seydel A., Camoirano A., Saccà S. C., Sandri M., Izzotti A. (2014). Oxidative damage and autophagy in the human trabecular meshwork as related with ageing. *PLoS One*.

[45] Zhou L., Li Y., Yue B. Y. (1999). Oxidative stress affects cytoskeletal structure and cell-matrix interactions in cells from an ocular tissue: the trabecular meshwork. *Journal of Cellular Physiology*.

[46] Lei Y., Stamer W. D., Wu J., Sun X. (2013). Oxidative stress impact on barrier function of porcine angular aqueous plexus cell monolayers. *Investigative Ophthalmology & Visual Science*.

[47] Fanning A. S., Ma T. Y., Anderson J. M. (2002). Isolation and functional characterization of the actin binding region in the tight junction protein ZO-1. *FASEB Journal : Official Publication of the Federation of American Societies for Experimental Biology*.

[48] Tornavaca O., Chia M., Dufton N. (2015). ZO-1 controls endothelial adherens junctions, cell-cell tension, angiogenesis, and barrier formation. *The Journal of Cell Biology*.

[49] Conlin V. S., Wu X., Nguyen C. (2009). Vasoactive intestinal peptide ameliorates intestinal barrier disruption associated with Citrobacter rodentium-induced colitis. *American Journal of Physiology Gastrointestinal and Liver Physiology*.

[50] Morampudi V., Conlin V. S., Dalwadi U. (2015). Vasoactive intestinal peptide prevents PKC*ε*-induced intestinal epithelial barrier disruption during EPEC infection. *American Journal of Physiology Gastrointestinal and Liver Physiology*.

[51] Xu J., Jia X., Gu Y., Lewis D. F., Gu X., Wang Y. (2017). Vitamin D reduces oxidative stress-induced procaspase-3/ROCK1 activation and MP release by placental trophoblasts. *The Journal of Clinical Endocrinology and Metabolism*.

[52] Akanda M. R., Kim M. J., Kim I. S. (2018). Neuroprotective effects of Sigesbeckia pubescens extract on glutamate-induced oxidative stress in HT22 cells via downregulation of MAPK/caspase-3 pathways. *Cellular and Molecular Neurobiology*.

[53] Yao H., Tang X., Shao X., Feng L., Wu N., Yao K. (2007). Parthenolide protects human lens epithelial cells from oxidative stress- induced apoptosis via inhibition of activation of caspase-3 and caspase-9. *Cell Research*.

[54] Onoue S., Endo K., Ohshima K., Yajima T., Kashimoto K. (2002). The neuropeptide PACAP attenuates *β*-amyloid (1-42)-induced toxicity in PC12 cells. *Peptides*.

[55] Onoue S., Ohmori Y., Endo K., Yamada S., Kimura R., Yajima T. (2004). Vasoactive intestinal peptide and pituitary adenylate cyclase-activating polypeptide attenuate the cigarette smoke extract-induced apoptotic death of rat alveolar L2 cells. *European Journal of Biochemistry*.

[56] Chen C. J., Ou Y. C., Li J. R. (2014). Infection of pericytes in vitro by Japanese encephalitis virus disrupts the integrity of the endothelial barrier. *Journal of Virology*.

[57] Chang C. Y., Li J. R., Chen W. Y. (2015). Disruption of in vitro endothelial barrier integrity by Japanese encephalitis virus-infected astrocytes. *Glia*.

[58] Zhang S., An Q., Wang T., Gao S., Zhou G. (2018). Autophagy- and MMP-2/9-mediated reduction and redistribution of ZO-1 contribute to hyperglycemia-increased blood-brain barrier permeability during early reperfusion in stroke. *Neuroscience*.

[59] Fong J. T., Kells R. M., Gumpert A. M., Marzillier J. Y., Davidson M. W., Falk M. M. (2012). Internalized gap junctions are degraded by autophagy. *Autophagy*.

